# The shortest innovative process for enhancing the S-allylcysteine content and antioxidant activity of black and golden garlic

**DOI:** 10.1038/s41598-022-15635-3

**Published:** 2022-07-07

**Authors:** Peeraporn Pakakaew, Yuthana Phimolsiripol, Siraphat Taesuwan, Sarawut Kumphune, Wannaporn Klangpetch, Niramon Utama-ang

**Affiliations:** 1 Division of Food Science and Technology, Faculty of Agro-Industry, Chiang Mai University, Chiang Mai, 50100 Thailand; 2 Division of Product Development Technology, Faculty of Agro-Industry, Chiang Mai University, Chiang Mai, 50100 Thailand; 3 Cluster of Innovative Food and Agro-Industry, Chiang Mai University, Chiang Mai, 50100 Thailand; 4 Biomedical Engineering Institute, Faculty of Engineering, Chiang Mai University, Chiang Mai, 50200 Thailand; 5 Cluster of High Value Product of Thai Rice and Plants for Health, Chiang Mai University, Chiang Mai, 50100 Thailand

**Keywords:** Biochemistry, Chemistry

## Abstract

Black garlic is a type of heat-treated garlic for which the traditional process is extremely simple yet time-consuming, taking more than one month. The purpose of this research was to reduce the processing time of black garlic while maintaining a high level of S-allylcysteine (SAC), a black garlic quality indicator. The fresh garlic was pre-treated with CaCl_2_ and frozen before being further incubated at two different temperatures (60 and 80 °C) with a relative humidity of 65% and 80% RH. Results showed that sequential pre-treatment and incubation at 80 °C and 80% RH for 1 week yielded 874.26 mg of SAC/100 g dry weight with an antioxidant activity of 5390 and 25,421 mg Trolox/100 g for DPPH and ABTS assays, respectively. This process shortened the processing time of black garlic by about 4-times. The batch processed at 60 °C and 65% RH for 1 week provided the highest SAC content of about 1772 mg/100 g dry weight, which was 2-times higher than in incubation at 80 °C and 80% RH for 1 week. The colour of this garlic was golden, so we call this new processed garlic product “golden garlic”.

## Introduction

Garlic is widely used as a seasoning for food and as a medicinal agent for the treatment of multiple human diseases and disorders because it has shown antimicrobial, antioxidant, anti-atherosclerotic and anti-cancer properties from the effects of its many active components. Garlic contains various bioactive compounds, such as organosulphur compounds, saponins, phenolic compounds and polysaccharides^1^. Sulphur-containing allicin (diallyl thiosulphinate) is the most well-known biologically active component in freshly crushed garlic cloves or extracts^2^. Allicin is made from the non-protein amino acid alliin by the enzyme alliinase^3^. The amount of thiosulphinates, which can affect garlic odour intensity and antibiotic properties, varies among garlic cultivars^4^. Although garlic has many active components that contribute to its health benefits, including allicin and its derivatives, the consumption of raw garlic has limits, because eating it excessively can cause stomach discomfort, and some people do not like its pungent flavour. At present, heat treatment is used to help improve the flavour of garlic to eliminate the unpleasant odour. In addition, heat increases the stability of the bioactive compounds in garlic^5^.

Black garlic is processed garlic produced by heat treatment under controlled humidity. Generally, it takes 1–2 months to produce without pre-treatment. During heat treatment, the colour of the garlic changes from white to black due to a browning process, and the total soluble solids content is improved, resulting in a sweet taste and a rubbery texture. It also has increased polyphenol content and antioxidant capacity^6^. This increased antioxidant capacity is also due to the formation of S-allylcysteine (SAC)^7^. SAC is formed by the enzymatic hydrolysis of γ-glutamyl-S-allylcysteine (GSAC), catalysed by γ-glutamyl transpeptidase (γ-GTP, EC 2.3.2.2). The SAC content is up to 6 times higher after the ageing process^8^. However, heat treatment reduces the activity of γ-GTP, which also results in reduced SAC formation. Therefore, temperature is one of the most important factors affecting the quality of black garlic during the manufacturing process.

The traditional process is time-consuming because the whole bulb of raw garlic is heated at high temperatures and controlled relative humidity without any pre-treatment. The high temperature destroys the cell structure and causes substances to react with each other^9^. Freezing has also been found to be a cell disruption method causing freezing injury^10^. Li et al.^11^ found that freezing for 30 h had the greatest impact on reducing sugar content, and increasing the total phenolic content and 5-hydroxymethyl-2-furaldehyde (5-HMF) levels. The amount of SAC is considered an important indicator of the quality of black garlic. SAC levels both before and after black garlic production should be considered because SAC is responsible for the antioxidant, anticancer, antihepatopathic and neurotrophic activities of black garlic^8^. In a study on the effect of soaking on the γ-GTP activity and SAC content in garlic, Xu et al.^12^ found that endogenous γ-GTP in garlic increased 23 times and the SAC content increased 4 times after soaking in 10 mM CaCl_2_ solution at 10 °C for 72 h. Accordingly, SAC, 5-HMF and the antioxidant activity can be used as quality factors for black garlic products.

However, the two pre*-*treatment methods consisting of CaCl_2_ soaking and freezing have never been used together to produce black garlic. Furthermore, the classification of GSAC, SAC and 5-HMF of black garlic produced by this method has not been studied. In this study, in order to reduce the processing time, a two-step pre-treatment method (CaCl_2_ and freezing) was used and the ageing process at different temperatures and relative humidity conditions was compared. The optimum condition for higher content of SAC was selected based on the functionality of the obtained black garlic products.

## Results and discussion

### Effect of processing conditions on bioactive compounds of black garlic

**Figure 1:**
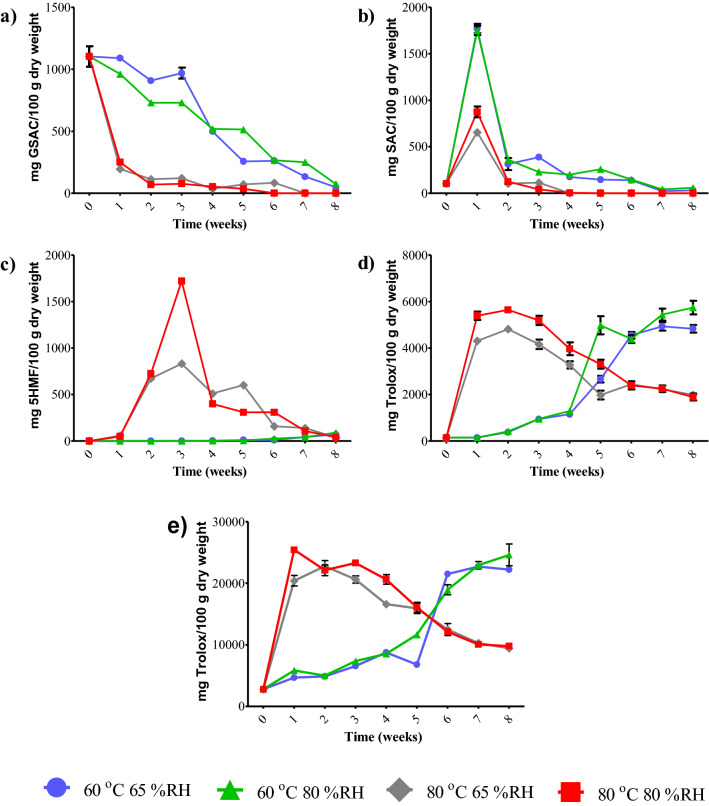
GSAC content (**a**), SAC content (**b**), 5-HMF content (**c**) and antioxidant activities by DPPH assay (**d**) and ABTS assay (**e**) of black garlic after different thermal processes at various temperatures and relative humidity; error bars represent standard deviations of the mean (n = 3).

The changes in the bioactive compounds including GSAC, SAC and 5-HMF of black garlic are reported in Table 1 and Fig. 1. The GSAC content (Fig. 1a) of black garlic produced at 80 °C decreased rapidly from 1104 to 250 mg/100 g dry weight in 1 week; meanwhile, for garlic produced at 60 °C, the GSAC content was slightly decreased. When GSAC content decreased, SAC content increased in week 1 (Fig. 1b). The SAC content of garlic produced at 60 °C was approximately 2-folds higher than that of garlic produced at 80 °C. The increase of SAC content was related to the increase of γ-GTP activity in the garlic, which is affected by the processing temperature^11^.

**Table 1:** Bioactive compounds of black garlic prepared using soaking followed by freezing pre-treatment methods at various temperatures and relative humidities.

Bioactive compounds	Treatments	Incubation time (weeks)								
		0	1	2	3	4	5	6	7	8
GSAC	60 °C 65%RH	1104.44 ± 82.14^A^	1090.06 ± 5.62^aA^	908.99 ± 3.06^aC^	969.01 ± 44.74^aB^	499.07 ± 10.72^bD^	256.48 ± 15.10^bE^	261.38 ± 4.95^aE^	134.03 ± 1.36^bF^	47.74 ± 0.19^bG^
	60 °C 80%RH	1104.44 ± 82.14^A^	961.58 ± 1.43^bB^	730.35 ± 2.58^bC^	730.70 ± 3.42^bC^	518.41 ± 0.60^aD^	513.98 ± 16.50^aD^	266.45 ± 8.70^aE^	250.52 ± 7.29^aE^	74.56 ± 0.30^aF^
	80 °C 65%RH	1104.44 ± 82.14^A^	195.55 ± 14.70^ dB^	112.24 ± 16.71^cC^	121.12 ± 13.71^cC^	35.03 ± 0.44^dD^	71.65 ± 6.90^cCD^	83.74 ± 5.24^bCD^	ND	ND
	80 °C 80%RH	1104.44 ± 82.14^A^	250.28 ± 0.62^cB^	68.89 ± 3.13^dC^	76.46 ± 2.10^dC^	53.17 ± 2.32^cC^	35.12 ± 0.84^dC^	ND	ND	ND
SAC	60 °C 65%RH	104.34 ± 10.38^F^	1772.15 ± 48.98^aA^	313.22 ± 63.75^aC^	387.42 ± 17.25^aB^	174.65 ± 9.65^bD^	146.39 ± 7.27^bE^F	138.78 ± 1.24^bEF^	22.25 ± 1.94^bG^	30.88 ± 1.73^bG^
	60 °C 80%RH	104.34 ± 10.38^E^	1750.29 ± 49.63^aA^	360.00 ± 33.43^aB^	228.79 ± 31.94^bCD^	200.54 ± 6.19^aD^	259.09 ± 22.56^aC^	145.71 ± 0.41^aE^	41.94 ± 4.51^aF^	58.03 ± 0.07^aF^
	80 °C 65%RH	104.34 ± 10.38^B^	654.50 ± 22.95^cA^	104.24 ± 14.77^bB^	113.43 ± 2.197^cB^	ND	ND	ND	ND	ND
	80 °C 80%RH	104.34 ± 10.38^B^	874.26 ± 57.27^bA^	123.97 ± 14.19^bB^	41.53 ± 4.49^dC^	4.34 ± 0.09^cD^	ND	ND	ND	ND
5-HMF	60 °C 65%RH	ND	ND	ND	1.81 ± 0.05^cF^	2.34 ± 0.02^dE^	11.58 ± 0.04^cC^	11.10 ± 0.02^dD^	42.69 ± 0.42^cB^	70.06 ± 0.03^bA^
	60 °C 80%RH	ND	ND	ND	1.19 ± 0.06^cF^	3.20 ± 0.03^cE^	5.56 ± 0.06^dD^	25.29 ± 0.03^cC^	41.35 ± 1.05^ dB^	89.45 ± 0.22^aA^
	80 °C 65%RH	ND	57.44 ± 0.29^aG^	673.41 ± 7.62^bB^	832.13 ± 1.46^bA^	511.24 ± 0.88^aD^	600.86 ± 3.95^aC^	158.79 ± 3.56^bE^	139.37 ± 0.06^aF^	38.69 ± 0.58^cH^
	80 °C 80%RH	ND	50.03 ± 0.04^bF^	724.60 ± 0.95^aB^	1721.41 ± 4.17^aA^	400.10 ± 0.23^bC^	309.40 ± 1.05^bD^	308.99 ± 0.79^aD^	107.47 ± 0.36^bE^	40.48 ± 2.32^cG^

^a–d^ Different superscripts within the same column indicate significant differences of each colour parameter compared for the same incubation time (*p* ≤ 0.05).

^A–G^Different superscripts within the same row indicate significant differences (*p* ≤ 0.05).

Garlic samples heated at low temperatures (60 °C) had more SAC content than at high temperatures (80 °C) because low temperature can promote the transformation of GSAC to SAC by γ-GTP better than at high temperature^13^. This agreed with the previously reported findings of Ahmed & Wang^13^ that the optimum temperature for γ-GTP activity was 40 °C. In addition, SAC formation is also affected by the water-facilitated reaction between GSAC and γ-GTP^8^ because the water in garlic assists the γ-glutamyl transferase reaction, assisting hydrolysis, and adequate water is need to support the transformation of GSAC into SAC^14^. High temperature showed greater water loss of garlic samples, resulting in γ-GTP being less active than at low temperature^8^. Therefore, the garlic sample heated at 60 °C had a higher SAC content than that heated at 80 °C.

After 2 weeks, the SAC content of garlic produced at 60 °C decreased fourfold; meanwhile, that of garlic produced at 80 °C decreased 6–7-fold when compared to week 1. The SAC gradually decreased until it could not be detected due to the acceleration of Maillard-type reactions between SAC and D-glucose^15^. Kimura et al.^16^ reported that the main volatile compounds formed by the Maillard reaction from the equimolar mixture of SAC and D-glucose were dimethyl disulphide, dimethyl trisulphide, dimethyl tetrasulphide, allyl methyl sulphide and an unknown compound with a molecular weight of m/z 162. However, in the presence of excess glucose, dimethyl disulphide, dimethyl trisulphide and dimethyl tetrasulphide were not formed from SAC^16^.

The results for 5-HMF content presented in Fig. 1c show that 5-HMF was not detected at week 0, but there was an increase in 5-HMF after heat treatment. In garlic produced at 80 °C, HMF increased rapidly during weeks 1–3 (50.03 to 1721.41 mg/100 g dry weight); meanwhile, 5-HMF was detected in week 3 in garlic produced at 60 °C (1.19–1.81 mg/100 g dry weight). 5-HMF is considered the most important intermediate product that occurs during two reactions: the acid-catalysed degradation of hexose and the decomposition of 3-deoxyosone in the Maillard reaction^17^. 5-HMF is produced under acidic conditions^18^. Therefore, when the pH decreased with increasing incubation time, 5-HMF increased. This is consistent with the research of Nakagawa et al.^19^, who found that the 5-HMF level of garlic heated at 70 °C increased as the number of heating days increased. In addition, extensive research reported that 5-HMF has antioxidant activity, anti-ischemic functions, and other beneficial effects on the human body^20^. It is noted that there was a continued increase of HMF in garlic produced at 60 °C, but in garlic produced at 80 °C, there was a continuous decrease in HMF after week 3, possibly due to moisture content^21^.

### Effect of processing conditions on antioxidant activity of black garlic

In terms of antioxidant activity (Table 2), both DPPH and ABTS assays showed a similar trend of changes in antioxidant activity (Fig. 1e,d). The antioxidant activity of garlic produced at 60 °C tended to increase with curing duration, possibly due to the representative antioxidant compounds in garlic: phenolics, flavonoids and sulphur-containing compounds such as SAC^22,23^. SAC plays a role in the antioxidant activity of black garlic by suppressing free radical formation^8^. The presence of browning reaction products (i.e. melanins, 5-HMF) also showed antioxidant abilities^24,25^. The antioxidant activity of garlic produced at 80 °C was increased dramatically in week 1 and was highest at week 2, after that it declined steadily. These effects can be primarily caused by temperature.

**Table 2:** Antioxidant activities of black garlic prepared using soaking followed by freezing pre-treatment methods at various temperatures and relative humidities.

Antioxidant activities	Treatments	Incubation time (weeks)								
		0	1	2	3	4	5	6	7	8
DPPH	60 °C 65%RH	146.96 ± 36.09^G^	146.29 ± 25.72^cG^	374.55 ± 25.80^cF^	944.95 ± 35.92^cE^	1153.14 ± 44.61^cD^	2661.51 ± 141.15^cC^	4554.27 ± 140.72^bB^	4935.68 ± 181.44^bA^	4831.98 ± 162.70^bA^
	60 °C 80%RH	146.96 ± 36.09^F^	150.11 ± 15.98^cF^	413.07 ± 23.97^cF^	953.70 ± 36.60^cE^	1290.14 ± 61.62^cD^	4980.54 ± 387.78^aB^	4388.32 ± 168.31^bC^	5439.41 ± 258.95^aA^	5744.09 ± 291.66^aA^
	80 °C 65%RH	146.96 ± 36.09^F^	4308.06 ± 114.87^bB^	4814.81 ± 127.55^bA^	4163.51 ± 205.50^bB^	3275.61 ± 154.13^bC^	1978.19 ± 190.85^dE^	2443.82 ± 74.17^aD^	2227.23 ± 135.20^cDE^	1983.14 ± 137.94^cE^
	80 °C 80%RH	146.96 ± 36.09^G^	5390.02 ± 180.03^aAB^	5643.58 ± 61.98^aA^	5194.55 ± 197.98^aB^	3969.23 ± 275.51^aC^	3306.00 ± 191.00^bD^	2393.74 ± 171.69^aE^	2247.79 ± 140.89^cE^	1891.82 ± 149.41^cF^
ABTS	60 °C 65%RH	2768.94 ± 176.53^G^	4681.05 ± 338.07^dF^	4862.37 ± 15.73^bF^	6564.28 ± 91.96^dE^	8753.91 ± 200.25^cD^	6778.47 ± 246.31^cE^	21,515.87 ± 234.81^aC^	22,720.39 ± 135.07^aA^	22,210.16 ± 312.42^bB^
	60 °C 80%RH	2768.94 ± 176.53^G^	5836.87 ± 151.82^cF^	4972.23 ± 271.51^bF^	7350.95 ± 151.56^cE^	8534.50 ± 433.20^cE^	11,646.24 ± 278.63^bD^	18,944.37 ± 818.73^bC^	22,918.52 ± 589.72^aB^	24,598.13 ± 1764.03^aA^
	80 °C 65%RH	2768.94 ± 176.53^F^	20,405.95 ± 858.88^bB^	22,759.16 ± 912.09^aA^	20,614.96 ± 598.57^bB^	16,611.14 ± 312.57^bC^	15,929.89 ± 846.49^aC^	12,485.74 ± 961.97^cD^	10,283.30 ± 497.19^bE^	9431.96 ± 321.73^cE^
	80 °C 80%RH	2768.94 ± 176.53^H^	25,421.11 ± 262.39^aA^	22,112.16 ± 856.43^aC^	23,300.72 ± 278.24^aB^	20,644.76 ± 776.78^aD^	16,091.93 ± 794.28^aE^	12,133.25 ± 326.23^cF^	10,053.47 ± 472.52^bG^	9797.35 ± 323.97^cG^

^a–d^ Different superscripts within the same column indicate significant differences of each colour parameter compared for the same incubation time (*p* ≤ 0.05).

^A–G^Different superscripts within the same row indicate significant differences (*p* ≤ 0.05).

Although the SAC and browning reaction products were formed during the production of black garlic, high temperatures can destroy the bioactive compounds contained in black garlic^26,27^. Therefore, the production of black garlic at 80 °C should only take 1–2 weeks to incubate for its high antioxidant activity. In addition, it can be seen that the antioxidant activity analysed by ABTS assay was higher than the DPPH assay, because the ABTS radical chromogens of the ABTS assay can be dissolved in both aqueous phases and organic phases; whereas DPPH assay uses a radical dissolved only in organic media^28^. Black garlic contains both hydrophilic and hydrophobic compounds^29^, resulting in a higher antioxidant activity by the ABTS assay.

### Effect of processing conditions on colour of black garlic

**Figure 2:**
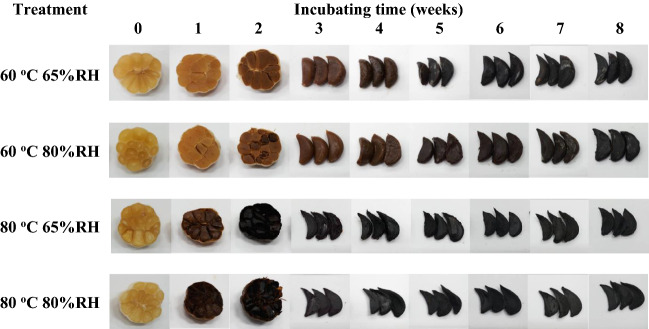
Black garlic prepared using CaCl_2_ soaking following freezing (SF) pre-treatment methods at various temperatures and relative humidities.

The colour results for black garlic produced at various temperatures (60 and 80 °C) and relative humidities (65 and 80% RH) showed that temperature had effect on colour (Fig. 2). During heat treatment, garlic changed colour from white/yellow to brown and black. The colour of garlic at higher temperature showed more change than at lower temperature due to a greater extent of Maillard reactions^8^. In addition, it can be seen that during weeks 2–8, black garlic treated with 80 °C was tougher, drier and harder than black garlic treated with 60 °C because high temperature accelerates water loss by excessive water evaporation^30^. Therefore, the garlic samples were charcoal-like after 2 weeks of processing at 80 °C and 5 weeks of processing at 60 °C.

**Figure 3:**
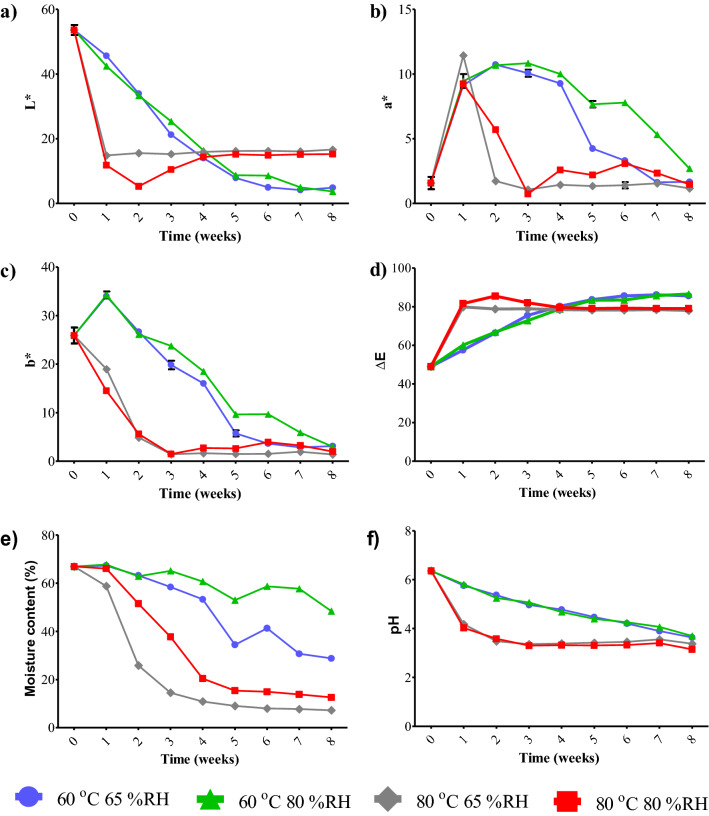
Colour of black garlic after different thermal processes at various temperatures and relative humidities: (**a**) L*, (**b**) a*, (**c**) b*, (**d**) ∆E, (**e**) moisture content and (**f**) pH; error bars represent standard deviations of the mean (n = 3).

L* decreased, but ∆E increased with increasing temperature. At 80 °C, L* decreased rapidly over 2 weeks; after that, it began to stabilise. Meanwhile, the 60 °C treatment caused a gradual decrease in L* (Fig. 3a). The a* of garlic produced at 60 °C increased slightly for 2–3 weeks and then showed a gradual decline; while after 1 week at 80 °C, a* dropped sharply and remained stable after week 3 (Fig. 3b). The b* change is similar to the L* change (Fig. 3c). The ∆E of garlic produced at 80 °C increased rapidly to more than 80 within 1 week then began to stabilise; while the ∆E of garlic produced at 60 °C was 80 after 4 weeks (Fig. 3d).

Black garlic is a processed garlic product formed by heat treatment under controlled humidity. The heating process leads to the Maillard reaction^31^ due to a chemical reaction between reducing sugars and the amino group in raw garlic. The main constituents of the polysaccharides and amino acids in raw garlic are fructan and arginine^32^. The Maillard reaction causes a colour change from the white of raw garlic to the dark brown of black garlic^5^ due to the formation of 5-HMF, melanoidin and other brown polymer compounds during heat treatment^19^. The decrease in a* and b* values with an increase in temperature and treatment time affects the colour change from yellow–brown to the red–black of black garlic. This indicates that there is an aggregation of melanoidin particles over time in black garlic produced at high temperatures^33^. Hence, L* decreases while ΔE increases during heat treatment. The ∆E increases very rapidly with an increase in temperature due to an increase in the reactivity of the Maillard reaction^34^.

### Effect of processing conditions on moisture content and pH of black garlic

The results from this experiment show that temperature had a significant effect on the colour values, while relative humidity did not affect the colour values. However, relative humidity did affect the black garlic’s moisture content (Fig. 3e). The moisture content of the black garlic produced at 80% RH was higher than that produced at 65% RH at the same temperature. In addition, the black garlic produced at the same relative humidity had a lower moisture content when produced at 80 °C than at 65 °C. This indicates that the moisture content of black garlic is affected by heat treatment conditions.

At higher temperatures, the moisture in the product decreases more rapidly, while a higher relative humidity during the process results in a higher moisture content. Initially, the decrease in moisture in the processed product proceeds slowly due to the high relative humidity, and then it proceeds very quickly due to evaporation at a high temperature^35,36^, after which it can slow down again or even stop due to the degradation of water-retaining compounds, such as fructans, which decompose into simple sugars or disaccharides during the garlic ageing process^37^. A decrease in moisture content affects the Maillard reaction because moisture is associated with limited molecular mobility and the consequent retarding of the reaction rate^38^. As previously reported by Van Boekel^21^, starting the Maillard reaction with a high water activity and then lowering it results in increasingly reactant concentrations. Therefore, the rate of Maillard reactions in our experiment increased at the initial stage. However, over time, the system will become too concentrated, resulting in less diffusion. Therefore, the Maillard reaction rate in our experiment decreased due to the reactants not easily meeting anymore. In this study, L* began to stabilise as the sample moisture content dropped below 40%.

During processing, pH was directly related to L* and b* but inversely proportional to ∆E because of the browning process. At the end of the process, after the garlic had turned perfectly black (∆E = 80)^39^ within 1 week at 80 °C and within 5 weeks at 60 °C, the pH of black garlic decreased to below 4.6 (Fig. 3f). A lower pH was found for black garlic, possibly due to the presence of acetic acid and succinic acid. Liang et al.^40^ observed acetic acid and succinic acid in black garlic, but not in fresh garlic samples. This is because during heat treatment, there is sugar fragmentation from the α-dicarbonyl and β-dicarbonyl of hexose or pentose to the short-chain carboxylic acid, which can also produce formic acid, succinic acid and 3-hydroxypropionic acid^40^. In addition, the decrease in pH can be associated with browning substances formed because of the formation of carboxylic acid substances^41^. A lower pH means that black garlic is more acidic^30^.

### Principal component analysis of black garlic qualities during heat treatment

**Figure 4:**
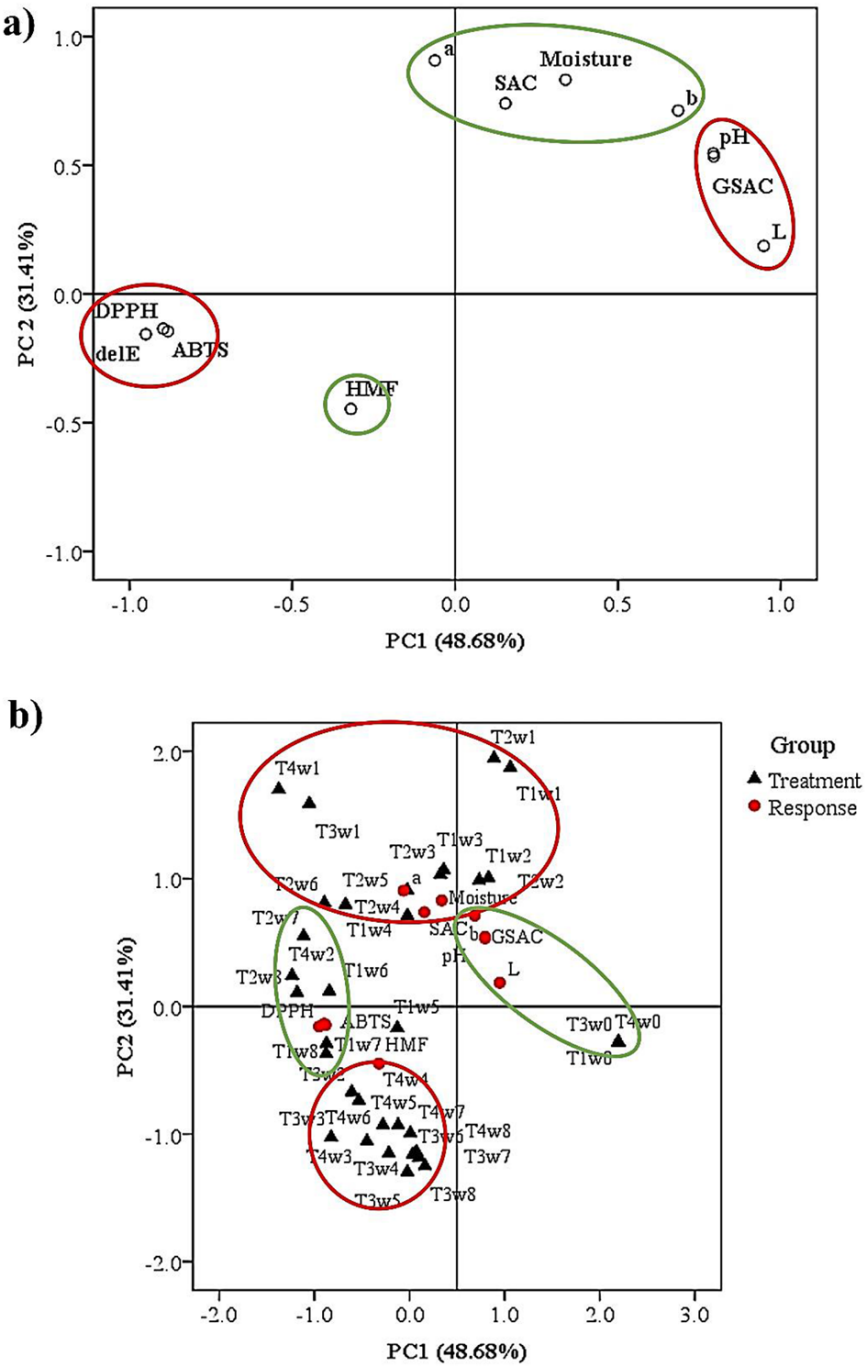
Principal component (PC) analysis of garlic qualities (**a**) and biplot analysis of garlic samples and their physiochemical properties (**b**); T1 = 60 °C and 65% RH, T2 = 60 °C and 80% RH, T3 = 80 °C and 65% RH, T4 = 80 °C and 80% RH and w0–w8 = weeks.

In this study, PCA was used to explain relationships between components of the black garlic sample. The PCA extracted two components with Eigen values greater than 1, explaining 80.09% of the total variance in the data set. The first and second components explained 48.68% and 31.41%, respectively, of all variations (Fig. 4a). The loadings express how well the new abstract principal components correlate with the old variables^42^. The first new abstract principal component, PC1, correlated well with the L*, pH and GSAC content of black garlic qualities. The ∆E, DPPH and ABTS were negatively correlated with the new PC. The second component, PC2, correlated well with the a*, b*, moisture content and SAC content. HMF was negatively correlated with the new PC.

The factor scores of each garlic sample on PC1 and PC2 were plotted on the regions defined by PC1 and PC2, resulting in a biplot showing the position of each garlic sample on PC1 and PC2 as shown in Fig. 4b. The data were categorised into four groups; the first group consists of samples treated with low temperature (60 °C) between 1–4 weeks that was distinct from the others by a*, b*, moisture and SAC contents. The second group consists of samples treated with low temperature (60 °C) between 6–8 weeks that was distinct from the others by $\Delta$E and antioxidant activity, and the third group consists of samples treated with high temperature (80 °C) that was distinct from the others by 5-HMF contents. Meanwhile, fresh garlic was separated and distinct from the others by L* and pH.

The fresh garlic sample was separated by L* and GSAC because it had white colour and high GSAC content. GSAC is often found in fresh garlic because it is a precursor to SAC formed during heat treatment^8^. Therefore, after heat treatment, SAC increases but GSAC decreases, resulting in high SAC contents of samples treated with low temperature (60 °C) between 1–4 weeks. However, high temperature and a long time process resulted in a decrease in SAC while 5-HMF increased due to the Maillard reaction. Therefore, samples treated at low temperature (60 °C) between 6–8 weeks changed in colour from white to black, resulting in high $\Delta$E and high antioxidant activity due to Maillard products, but low SAC contents. In addition, samples treated with high temperatures (80 °C) had higher 5-HMF content but lower moisture content than samples treated with low temperatures, because high temperatures increase the rate of Maillard reactions^34^ and accelerate the evaporation of water more than low temperatures^37^.

On the contrary, an increase in the acidity during the process due to the browning reaction also reduced the pH of the black garlic. It dropped below 4.2 after the first week of aging due to carboxylic acids that formed during thermal processing by a browning reaction^39^. The effect of high temperature and acidity may inhibit γ-GTP activity because the optimum pH and temperature for γ-GTP activity are pH 6 and 70 °C^43^. Therefore, samples treated with high temperatures (80 °C) have less SAC content than those treated at low temperatures. For this reason, the samples can be divided into four main groups, namely, the fresh garlic group, samples treated at low temperature (60 °C) between 1–4 weeks, samples treated at low temperature (60 °C) between 6–8 weeks and samples treated with high temperature (80 °C).

### Comparison of bioactive compounds and antioxidant activity of various processed garlic

In this study, the most efficient condition for producing black garlic was 80 °C and 80% RH because this condition had a short processing time of within 1 week, and produced soft and elastic black garlic with a final moisture content of 66.03 ± 0.04% and ∆E of 81.60 ± 0.04. Even though the developed black garlic had a lower ∆E than commercial black garlic, it was more than 80, so it can be considered “perfect black garlic” as well^39^. In addition, the produced black garlic had high antioxidant activity of 5390.02 ± 180.03 and 25,421.11 ± 262.39 mg Trolox/100 g dry weight when analysed with DPPH and ABTS, respectively. However, the golden garlic that was produced under 60 °C and 65% RH for 1 week can provide the highest SAC content (1772.15 ± 48.98 mg/100 g dry weight) which was approximately 2 times more than black garlic produced in the experiment.

Therefore, it can be said that in this study, two methods of garlic processing were discovered: (1) black garlic processing and (2) golden garlic processing. Moreover, the comparison of the amount of SAC, 5-HMF and antioxidant activity in Table 3 (*p* ≤ 0.05) found that developed golden garlic had the highest SAC content which was 11 times higher than commercial black garlic, but it had the lowest antioxidant activity. Although SAC is the predominant antioxidant of black garlic, 5-HMF is one of the major antioxidant ingredients as well as another browning product from Maillard reaction. Therefore, black garlic had higher antioxidant activity than golden garlic^13^.

**Table 3:** Comparison of bioactive compounds and antioxidant activity of developed golden garlic, developed black and commercial black garlic.

	Developed golden garlic (60 °C 65%RH; 1 week)	Developed black garlic (80 °C 80%RH; 1 week)	Commercial black garlic (60 °C; 2 months)
**Bioactive compounds (mg/100 g dry weight)**			
GSAC content	1090.06 ± 5.62^a^	250.28 ± 0.62^b^	27.12 ± 1.06^c^
SAC content	1772.15 ± 48.98^a^	874.26 ± 57.27^b^	150.86 ± 8.14^c^
5-HMF content	–	50.03 ± 0.01^a^	9.85 ± 0.67^b^
**Reducing sugar (mg/100 g dry weight)**	69.72 ± 1.15^c^	634.92 ± 8.49^b^	783.85 ± 37.55^a^
**Antioxidant activity (mg TEAC/100 g dry weight)**			
DPPH assay	146.291 ± 25.72^c^	5390.02 ± 180.03^a^	3550.15 ± 65.72^b^
ABTS assay	4681.05 ± 338.07^c^	25,421.11 ± 262.39^a^	17,184.27 ± 436.30^b^
**Colour**			
L*	45.63 ± 0.57^a^	11.79 ± 0.08^b^	5.60 ± 0.03^c^
a*	9.13 ± 0.13^a^	9.25 ± 0.14^a^	5.80 ± 0.03^b^
b*	33.97 ± 0.11^c^	14.53 ± 0.15^b^	6.02 ± 0.05^a^
∆E	57.49 ± 0.47^c^	81.60 ± 0.04^b^	85.21 ± 0.03^a^
**Moisture content (%)**	67.25 ± 0.06^a^	66.03 ± 0.04^b^	49.35 ± 0.43^c^
**pH**	5.76 ± 0.06^a^	4.03 ± 0.02^b^	4.05 ± 0.02^b^

^a–^^c^Different superscripts within the same column indicate significant differences (*p* ≤ 0.05).

In addition, the black garlic in the experiment contained approximately 5 times higher SAC than commercial black garlic and had the highest 5-HMF and antioxidant activity. However, the commercial black garlic had the highest reduction of sugar, which was 11 times more reduced than the golden garlic. Because during heat treatment the enzymatic and thermal degradation of polysaccharides to glucose and fructose was catalysed by fructan EXOhydrolase^37^, the black garlic presented more sweetness than golden garlic^41^.

## Conclusion

This research showed the effect of the combined pre-treatment process of CaCl_2_ and freezing on black garlic processed at different temperatures and relative humidity conditions. The ideal condition for black garlic after the combined pre-treatment process was 80 °C and 80% RH. Under this condition, the incubation process was reduced to 1 week with SAC content of 874.26 ± 57.27 mg/100 g dry weight. The highest SAC content (1772 mg/100 g dry weight) was obtained at 60 °C and 65% RH. This produced a new processed garlic product was called “golden garlic” due to its golden colour. The presence of bioactive compounds including SAC and 5-HMF in black garlic might be beneficial for their therapeutic effects. In conclusion, the combined pre-treatment process might be a way to reduce the black garlic processing time and this information might be a useful tool for industrial application.

## Materials and methods

### Raw material and chemicals

Garlic (*Allium* *sativum* L.) was purchased from local markets in Srisaket province (Jae Mam Phanit), Thailand. Identification was done according to an Alliaceae expert and the literature^44^. A voucher specimen (GA55001) was referenced by the Srisaket Horticultural Research Centre, Srisaket, Thailand. The garlic samples were kept at room temperature until use. The use of plants in the present study complies with international, national and institutional guidelines.

2,2-Diphenyl-1-picrylhydrazyl (DPPH), 2,2′-azino-bis(3-ethylbenzothiazoline-6-sulfonic acid) diammonium salt (ABTS), S-allyl-L-cysteine (SAC), 5-(hydroxymethyl)furfural (5-HMF), 3,5-dinitrosalicylic acid (DNS) and D-glucose were purchased from Sigma-Aldrich (Massachusetts, USA). L-γ-Glutamyl-(S)-allyl-cysteine (GSAC) was purchased from Toronto Research Chemicals (Toronto, Canada). Anhydrous sodium carbonate, aluminium chloride and potassium acetate were purchased from Loba Chemie (Mumbai, India). Ethanol was purchased from RCI Labscan Ltd. (Bangkok, Thailand). All other chemicals and reagents used in the experiments were analytical grade.

### Processing conditions of black garlic

The fresh garlic was peeled and sized, and only clean whole cloves were selected. Selected fresh garlic bulbs were pre-treated by soaking with CaCl_2_ at 10 °C for 72 h, and then they were frozen at –18 °C for 30 h. After that, pre-treated garlic was heated at two temperatures (60 and 80 °C) in two relative humidities (65 and 80% RH) for 8 weeks in a drying oven (UF110, Memmert, Germany). Colour, pH, moisture content, GSAC, SAC, 5-HMF and antioxidant activity were determined every week for up to 8 weeks.

### Preparation of black garlic extracts

The black garlic was peeled and then ground in a blender (HR2115, Philips, Indonesia) and mixed with 50% ethanol at a ratio of 1: 10 (w/v). The mixture was extracted using the condition of Pakakaew et al.^45^ at 60 °C for 90 min in a water bath shaker (Memmert, Schwabach, Germany), filtered through Whatman® no. 4 filter paper, and the filtrate was kept at − 18 °C for analysis.

### GSAC, SAC and 5-HMF content measurements

The extracts were filtered through a 0.45-µL syringe filter before being injected into a high-performance liquid chromatograph (HPLC) with a UV detector (1260 Infinity II, Agilent Technologies, Santa Clara, CA, USA) to analyse the GSAC, SAC and 5-HMF contents. The HPLC column was an Inertsil ODS-3 (C18 column, 5 µm, 4.6 × 250 mm, GL Sciences Inc., Japan). The isocratic HPLC system was used for analysis. The mobile phase was prepared by mixing 50 mM phosphate buffer (pH 2.8) and methanol in a ratio of 85:15 v/v, respectively, all HPLC grade. The flow rate of the mobile phase was 1 mL/min, with a sample injection volume of 10 µL and column temperature of 25 °C. The detection wavelength was 205 nm for GSAC and SAC detection^46^ and 285 nm for 5-HMF detection^47^.

### Reducing sugar

Reducing sugar was determined by a modified method of Somjai et al.^48^. Glucose solutions (0.15–5.00 mg/mL) were plotted as a standard curve. One mL of extract and 4 mL of DNS reagent were mixed in a test tube and covered with aluminum foil before boiling in a water bath (90 °C) for 5 min. The test tube was immediately cooled, and 10 mL of distilled water was added. The absorbance of mixed samples was measured at 550 nm by a UV spectrophotometer (GENESYS 10S UV–Vis, Thermo Fisher Scientific, USA).

### DPPH radical scavenging activity

The DPPH radical scavenging activity was determined using the method of Lu et al.^49^ with minor modifications. For detection, 300 µL of garlic extract was mixed with 3000 µL of 0.208 mM DPPH solution. The mixture was then incubated for 30 min in the dark at room temperature followed by the measurement of its absorbance at 515 nm. Trolox was used as a standard at concentrations of 0–500 µmol/L. The antioxidant capacity of the sample was expressed as mg Trolox equivalent antioxidant capacity (TEAC) per 100 g dry weight. The measurements were carried out in triplicate.

### ABTS radical cation scavenging activity

The ABTS radical cation scavenging activity was modified from the method of Surin et al.^50^. Briefly, 7 mM ABTS was dissolved in distilled water with 2.45 mM potassium persulfate. The mixture was incubated 12–16 h in the dark at 30 °C to obtain ABTS radical cations (ABTS• +). The ABTS• + solution was adjusted with a sodium phosphate buffer (0.1 M, pH 7.4) to an initial absorbance of 0.75 ± 0.005 at 734 nm. For determination, 2900 µL of the ABTS• + solution was well mixed with 100 µL of garlic extract. The mixture was then incubated for 10 min in the dark at 25 °C followed by the measurement of its absorbance at 734 nm. Trolox was used as the calibrating standard. The antioxidant capacity of the sample was expressed as mg Trolox equivalent antioxidant capacity (TEAC) per 100 g dry weight. The measurements were carried out in triplicate.

### Colour and pH measurements

The colour values of the garlic samples were measured using a MiniScan EZ 4500L spectrophotometer (Hunter Associates Laboratory, Inc., Reston, Virginia, USA) calibrated with black and white porcelain reference plates following the method of Phimolsiripol et al.^51^. The CIE L*, a* and b* colour spaces were measured. L* indicates lightness, and a* and b* indicate colour directions; + a* is redness, − a* is greenness, + b* is yellowness and − b* is blueness. The pH was measured using an electronic pH meter (Mettler Toledo FE20, Küsnacht, Switzerland).

### Statistical analysis

All experiments were conducted in triplicate. The mean and standard error of the mean were determined using SPSS Statistics 17.0 for Windows (SPSS Inc., Chicago, IL, USA). Differences among the means of treatment were determined using analysis of variance (ANOVA) with Duncan’s multiple range post hoc analysis at the 95% confidence level. Principal component analysis (PCA) and biplots were also generated to analyse the correlation structure of a group of multivariate observations and provide the axis along which the maximum variability in the data occurred.

## References

[1] Shang A (2019). Bioactive compounds and biological functions of garlic (*Allium*
*sativum* L.). Foods.

[2] Bat-Chen W, Golan T, Peri I, Ludmer Z, Schwartz B (2010). Allicin purified from fresh garlic cloves induces apoptosis in colon cancer cells via Nrf2. Nutr. Cancer.

[3] Rachchapan, R. *et**al.* in *Proceedings**of**Innovation**and**Technology**Conference,**Surin,**Thailand.* 409–416.

[4] Camargo, A., Masuelli, R. & Burba, J. in *IV**International**Symposium**on**Edible**Alliaceae**688.* 309–312.

[5] Kang O-J (2016). Physicochemical characteristics of black garlic after different thermal processing steps. Prev. Nutr. Food Sci..

[6] Kim I-D, Park Y-S, Park J-J, Dhungana SK, Shin D-H (2019). Physicochemical and antioxidant properties of garlic (A. sativum) prepared by different heat treatment conditions. Korean J. Food Sci. Technol..

[7] Lee Y-M (2009). Antioxidant effect of garlic and aged black garlic in animal model of type 2 diabetes mellitus. Nurs. Res. Pract..

[8] Bae SE, Cho SY, Won YD, Lee SH, Park HJ (2014). Changes in S-allyl cysteine contents and physicochemical properties of black garlic during heat treatment. LWT-Food Sci. Technol..

[9] Hu S, Ding Y, Zhu C (2020). Sensitivity and responses of chloroplasts to heat stress in plants. Front. Plant Sci..

[10] Shehadul Islam M, Aryasomayajula A, Selvaganapathy PR (2017). A review on macroscale and microscale cell lysis methods. Micromachines.

[11] Li N, Lu X, Pei H, Qiao X (2015). Effect of freezing pretreatment on the processing time and quality of black garlic. J. Food Process Eng.

[12] Xu X, Miao Y, Chen JY, Zhang Q, Wang J (2015). Effective production of S-allyl-L-cysteine through a homogeneous reaction with activated endogenous γ-glutamyltranspeptidase in garlic (Allium Sativum). J. Food Sci. Technol..

[13] Ahmed T, Wang C-K (2021). Black garlic and its bioactive compounds on human health diseases: A review. Molecules.

[14] Ai TT, Huong NT (2018). Research on the production of black garlic juice. Int. J. Pharm. Sci. Invent..

[15] Wakamatsu J, Stark TD, Hofmann T (2016). Taste-active Maillard reaction products in roasted garlic (Allium sativum). J. Agric. Food Chem..

[16] Kimura K (1990). Thermal degradation products of S-Alkyl-l-cysteine occurring in the A Ilium species with d-glucose. Agric. Biol. Chem..

[17] Shapla UM, Solayman M, Alam N, Khalil M, Gan SH (2018). 5-Hydroxymethylfurfural (HMF) levels in honey and other food products: effects on bees and human health. Chem. Cent. J..

[18] Chen Y, Lin H, Li Y, Lin M, Chen J (2019). Non-enzymatic browning and the kinetic model of 5-hydroxymethylfurfural formation in residual solution of vinegar soaked-soybean. Ind. Crops Prod..

[19] Nakagawa K, Maeda H, Yamaya Y, Tonosaki Y (2020). Maillard reaction intermediates and related phytochemicals in black garlic determined by EPR and HPLC analyses. Molecules.

[20] Lu X, Li N, Qiao X, Qiu Z, Liu P (2017). Composition analysis and antioxidant properties of black garlic extract. J. Food Drug Anal..

[21] Van Boekel M (2001). Kinetic aspects of the Maillard reaction: a critical review. Food Nahrung.

[22] Leelarungrayub N, Rattanapanone V, Chanarat N, Gebicki JM (2006). Quantitative evaluation of the antioxidant properties of garlic and shallot preparations. Nutrition.

[23] Sunanta P (2021). Does curing moisture content affect black garlic physiochemical quality?. Horticulturae.

[24] Eichner K (1980). In: Autoxidation in food and biological systems.

[25] Zhao L (2013). In vitro antioxidant and antiproliferative activities of 5-hydroxymethylfurfural. J. Agric. Food Chem..

[26] Rahim MS, Salihon J, Yusoff MM, Bakar IA, Damanik MR (2010). Effect of temperature and time to the antioxidant activity in *Plecranthus*
*amboinicus* Lour. Am. J. Appl. Sci..

[27] Réblová Z (2012). Effect of temperature on the antioxidant activity of phenolic acids. Czech J. Food Sci..

[28] Kim D-O, Lee KW, Lee HJ, Lee CY (2002). Vitamin C equivalent antioxidant capacity (VCEAC) of phenolic phytochemicals. J. Agric. Food Chem..

[29] Kwartika O (2020). A review: Antioxidant and immunomodulator effects of black garlic. EAS J. Pharm. Pharmacol..

[30] Bedrníček J (2021). The use of a thermal process to produce black garlic: Differences in the physicochemical and sensory characteristics using seven varieties of fresh garlic. Foods.

[31] Ryu JH, Kang D (2017). Physicochemical properties, biological activity, health benefits, and general limitations of aged black garlic: A review. Molecules.

[32] Kodera Y, Kurita M, Nakamoto M, Matsutomo T (2020). Chemistry of aged garlic: Diversity of constituents in aged garlic extract and their production mechanisms via the combination of chemical and enzymatic reactions. Exp. Ther. Med..

[33] Echavarría AP, Pagán J, Ibarz A (2016). Kinetics of color development in glucose/amino acid model systems at different temperatures. Sci. Agropecuaria.

[34] Arachchi SJT, Kim Y-J, Kim D-W, Oh S-C, Lee Y-B (2017). Optimization of Maillard reaction in model system of glucosamine and cysteine using response surface methodology. Prev. Nutr. Food Sci..

[35] Sun Y-E, Wang W (2018). Changes in nutritional and bio-functional compounds and antioxidant capacity during black garlic processing. J. Food Sci. Technol..

[36] Toledano Medina MÁ, Pérez-Aparicio J, Moreno-Ortega A, Moreno-Rojas R (2019). Influence of variety and storage time of fresh garlic on the physicochemical and antioxidant properties of black garlic. Foods.

[37] Najman K, Sadowska A, Hallmann E (2021). Evaluation of bioactive and physicochemical properties of white and black garlic (*Allium*
*sativum* L.) from conventional and organic cultivation. Appl. Sci..

[38] Gonzales AP, Naranjo G, Leiva G, Malec L (2010). Maillard reaction kinetics in milk powder: Effect of water activity at mild temperatures. Int. Dairy J..

[39] Zhang X, Li N, Lu X, Liu P, Qiao X (2016). Effects of temperature on the quality of black garlic. J. Sci. Food Agric..

[40] Liang T (2015). Comprehensive NMR analysis of compositional changes of black garlic during thermal processing. J. Agric. Food Chem..

[41] Zhang Z (2015). Evaluation of alliin, saccharide contents and antioxidant activities of black garlic during thermal processing. J. Food Biochem..

[42] Csomós E, Héberger K, Simon-Sarkadi L (2002). Principal component analysis of biogenic amines and polyphenols in Hungarian wines. J. Agric. Food Chem..

[43] Sun Y, Hu J, Wang W, Zhang B, Shen Y (2019). Characterization of γ-glutamyltranspeptidases from dormant garlic and onion bulbs. Food Sci. Nutr..

[44] Austin, J. *et**al.* Verification the identity of garlic from different growing area. *Thai**Agric.**Res.**J.**(Thailand)* (2016).

[45] Pakakaew P, Taesuwan S, Phimolsiripol Y, Utama-ang N (2022). Comparison between the physicochemical properties, bioactive compounds and antioxidant activities of Thai and Chinese Garlics. Curr. Appl. Sci. Technol..

[46] Ichikawa M, Ide N, Yoshida J, Yamaguchi H, Ono K (2006). Determination of seven organosulfur compounds in garlic by high-performance liquid chromatography. J. Agric. Food Chem..

[47] Kalábová L, Večerek V (2006). Hydroxymethylfurfural contents in foodstuffs determined by HPLC method. J. Food Nutr. Res..

[48] Somjai C (2022). Effect of drying process and long-term storage on characterization of Longan pulps and their biological aspects: Antioxidant and cholinesterase inhibition activities. LWT.

[49] Lu X (2011). Determination of total phenolic content and antioxidant capacity of onion (Allium cepa) and shallot (Allium oschaninii) using infrared spectroscopy. Food Chem..

[50] Surin S, Surayot U, Seesuriyachan P, You S, Phimolsiripol Y (2018). Antioxidant and immunomodulatory activities of sulphated polysaccharides from purple glutinous rice bran (Oryza sativa L). Int. J. Food Sci. Technol..

[51] Phimolsiripol Y (2021). Shelf life extension of chilled pork by optimal ultrasonicated Ceylon Spinach (Basella alba) extracts: Physicochemical and microbial properties. Foods.

